# A rare case of pancreatic head hydatid cyst

**DOI:** 10.1093/jscr/rjab243

**Published:** 2021-06-30

**Authors:** Muhammad M Qarmo, Alaa N Aldirani, Lynn M J Al-Boukhari, Faisal M Moussa, Lama A Alkhateeb

**Affiliations:** Department of General Surgery, General Assembly of Damascus Hospital, Al-Furat University, Damascus, Syria; Department of General Surgery, General Assembly of Damascus Hospital, Damascus, Syria; Department of General Surgery, General Assembly of Damascus Hospital, Damascus University, Damascus, Syria; Department of General Surgery, General Assembly of Damascus Hospital, Damascus, Syria; Department of General Surgery, General Assembly of Damascus Hospital, Damascus University, Damascus, Syria

**Keywords:** hydatid cyst, head of the pancreas, pancreaticoduodenectomy

## Abstract

Hydatidosis or Echinococcosis is a parasitic disease caused by infection with the larval stage of Echinococcus granulosus. Primary isolated pancreatic hydatidosis is very rare even in countries where echinococcosis disease is highly endemic. The objective of this case report is to highlight this unusual and rare hydatid cyst presentation to avoid incorrect diagnosis and management. In our case, preoperative evaluation guided the diagnosis toward cystic pancreatic neoplasms, leading us to contemplate a radical surgical approach (Pancreaticoduodenectomy). Pancreatic hydatid cysts can be confused with cystic pancreatic neoplasms, it should always be considered as a differential diagnosis in endemic areas, to prevent misdiagnosis and inappropriate management.

## INTRODUCTION

Hydatid disease is a parasitic disease caused by infection with the larval stage of Echinococcus granulosus. Theoretically, echinococcosis can involve any organ. The liver is the most common organ involved, followed by the lungs. These two organs account for 90% of cases of echinococcosis [1]. Pancreatic Hydatidosis remains rare even in countries where the disease is endemic, with an incidence ranging from 0.14 to 2% [2, 3, 4]. The diagnosis can be difficult, confusing with other cystic lesions of the pancreas. Signs and symptoms depend on the location, size of the enlarging cyst and pressure exerted on the surrounding tissues. Hydatid cysts may remain silent and asymptomatic or cause severe to life-threatening complications requiring prompt medical treatment.

Here, we present a case of a primary hydatid cyst located in the head of the pancreas causing dilatation of the main pancreatic duct.

## CASE PRESENTATION

**Figure 1:**
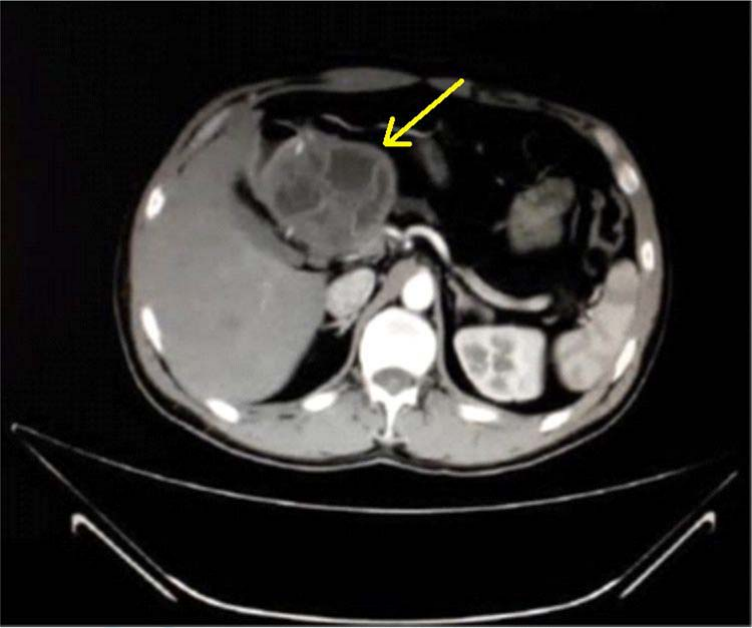
Axial contrast-enhanced CT scan of the abdomen demonstrating a well-defined solitary cystic lesion in the head of the pancreas with a diameter of (9 cm).

**Figure 2:**
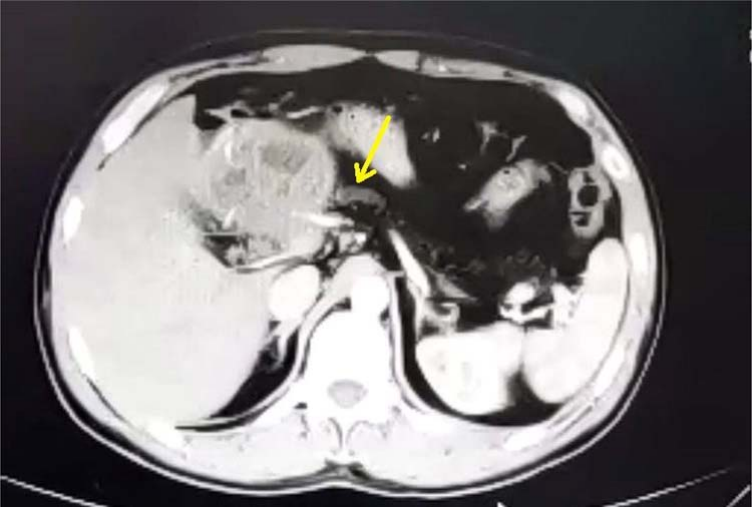
Axial contrast-enhanced CT scan of the abdomen showing dilatation of the MPD (5 mm).

A 33-year-old male was referred to our Department of General Surgery, with a 3-month severe epigastric pain radiating to the back, associated with several episodes of green vomiting. Pain is episodic, relieved by painkillers, has no relation to food or movement. No history of changes in bowel habits or urine color. The patient reported a history of early satiety and an observed unmeasured weight loss. The past medical and surgical histories were irrelevant. Abdominal examination showed tenderness in the epigastric region, with no other signs. Laboratory tests including complete blood cell count, renal and liver function tests were within normal levels. Immunologic methods (ELISA) and indirect hemagglutination (IHA) assays were negative. Serum Amylase and CA 19-9 were within normal levels. Computed tomography (CT) scan revealed a multilocular cystic lesion in the head of the pancreas measuring 9.5 × 8.2 × 11 cm, causing external compression on the distal part of the CBD. The main pancreatic duct near the cystic mass is dilated measuring 0.5 cm. The gallbladder is distended (Figs 1 and 2).

**Figure 3:**
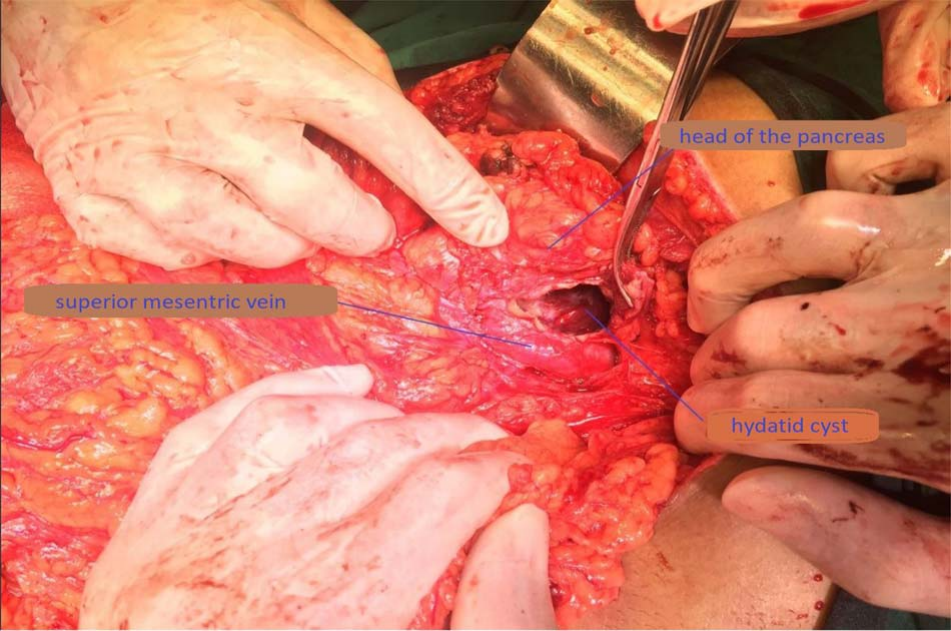
Operative photograph demonstrating the location of the unroofed cyst in the pancreatic head near the superior mesenteric vein.

Percutaneous US-guided fine-needle aspiration was performed and the result revealed malignant cells. Such findings orient the diagnosis toward Intraductal Papillary Mucinous Neoplasm (IPMN) and Mucinous Cystic Neoplasm (MCN). The patient was admitted to the hospital, and an exploratory laparotomy was carried out both to diagnose the condition and to perform the necessary therapeutic procedure. Intraoperatively, a cystic structure was found, located in the head of the pancreas and was adhered to the superior mesenteric vein (Fig. 3). The cyst was carefully separated from all adhesions and surrounding structures. Intra-operative needle aspiration of the cyst was repeated. On analysis, the clear colorless fluid demonstrated hydatid scolices and hooklets.

**Figure 4:**
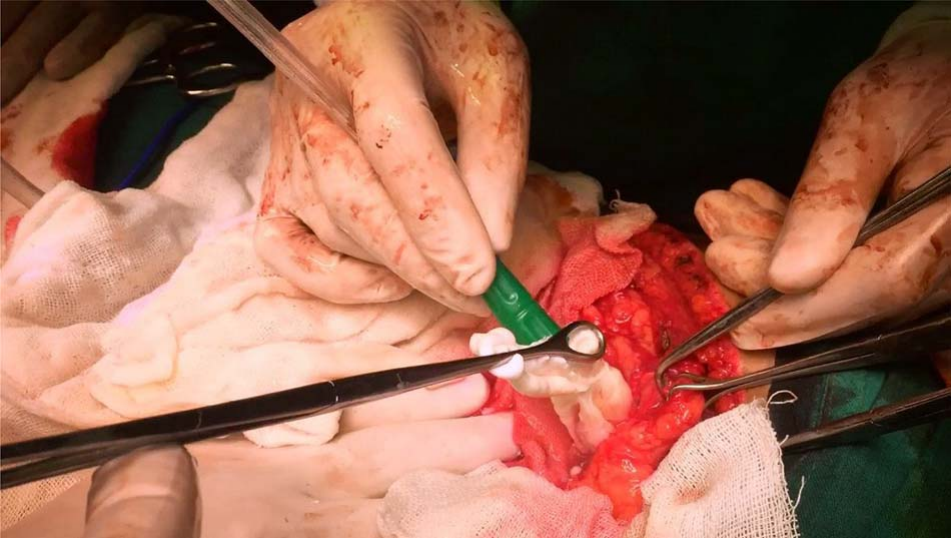
Operative photograph demonstrating the extraction of the germinative membrane.

The cystic fluid was aspirated, after that a scolicidal agent (hypertonic saline 20%) was instilled into the cyst cavity, then it was re-aspirated. The cyst was then opened, followed by evacuation of the germinative membrane and daughter vesicles (Fig. 4).

Careful examination of the cyst showed no communication with the pancreatic duct. Partial cystectomy was performed along with omentoplasty. A drain was placed in the residual cavity. Histopathological examination confirmed the diagnosis of pancreatic hydatid cyst (PHC). The post-operative course was uneventful and the patient was discharged on oral Albendazole therapy for 4 months. The drainage tube was removed 5 days later. During 6 months of follow-up, the patient was in good condition with no complications or recurrences.

## DISCUSSION

Hydatid disease is a parasitic infestation by the tapeworm Echinococcus granulosus in its larval stage. Hydatidosis is more predominant in sheep-raising communities, where dogs have access to infected offal. Human infection occurs by ingesting food or water contaminated with infected-dog feces containing viable tapeworm eggs.

PHCs, despite being rare, have been reported with an incidence of 0.14–2% [2, 3, 4]. They are commonly seen in the head of the pancreas (50–58%), followed by the body (24–34%) and the pancreatic tail (16–19%) [11]. Pancreatic infestation by Echinococcus granulosus occurs mainly by hematogenous dissemination [10]. Clinical presentation varies and depends on mass effect or cyst complications. Cysts located in the head can present as obstructive jaundice due to external compression of the common bile duct, and masquerade as a choledochal cyst [5, 6]. Cysts located in the body of the pancreas are usually asymptomatic until they become large enough to present as an abdominal lump or cause symptoms due to compression of adjacent structures. Rarely, cysts located in the pancreatic tail can result in splenomegaly, and portal hypertension. Complications such as cholangitis, rupture into the biliary tree or peritoneal cavity, pancreatic fistula, recurrent pancreatitis and abscess have also been described [7–9]. Cyst rupture into the peritoneal cavity is a rare but serious complication. The hydatid fluid is antigenic and highly toxic and can cause a potentially fatal anaphylactic reaction in humans.

Various imaging modalities are used in the diagnosis of hydatid disease such as ultrasonography, CT scan, MRI and endoscopic US. Accurate diagnosis cannot be established based on radiological findings alone, and this is especially true in pancreatic cystic lesions in which imaging features such as multilocular cysts, internal septations, calcifications and wall enhancement are encountered in both benign (hydatid cysts) and malignant (MCNs, serous cystadenoma, IPMN). In this report, our patient showed worrisome radiological features such as dilatation of the MPD.

Serological testing is a common method for the diagnosis of hydatid cysts. Enzyme-linked immunosorbent assay (ELISA) for echinococcal antigens results is positive in ~85% of infected patients with hepatic hydatidosis, whereas the seropositivity rate is 54% for PHC cases [11, 12]. ELISA results may be negative in an infected patient if the cyst has not leaked or does not contain scolices or if the parasite is no longer viable. Negative serology cannot be used to definitively exclude Cystic Echinococcosis. Endoscopic or Percutaneous US-guided FNA cytology is another diagnostic method used in the evaluation of cystic pancreatic lesions. The accuracy of FNA cytology in cystic pancreatic masses is 62% [13]. However, there are reports of falsely positive FNA interpretations of pancreatic cytology [14].

The occurrence of hydatid cysts in unusual abdominal sites presents a major diagnostic challenge, and this is particularly true for primary, isolated lesions with no lung or liver involvement. In addition to the previous features, our patient had alarming radiological findings (dilatation of the MPD), malignant result in the pre-operative FNA and negative serological testing. All of which were consistent with pancreatic cystic neoplasms. The preoperative FNA result represented a major dilemma in deciding whether to explore the cyst or not, as it carried the potential risk of peritoneal dissemination of viable neoplastic cells. However, the surgeon’s decision to obtain an intra-operative FNA of the cystic lesion was of great value. The outcome had an enormous impact on the course of management, by preventing major procedures with high morbidity and mortality rates such as Pancreaticoduodenectomy, as well as adopting a safer and less invasive surgical approach.

While surgery remains the only definitive diagnostic and therapeutic tool in the management of PHC [7, 8], a variety of treatment modalities have been described, including medical therapy, open or laparoscopic surgical approach and minimally invasive approach: direct percutaneous catheterization or PAIR (puncture, aspiration, injection, re-aspiration). The appropriate surgical procedure depends on several factors such as the localization and size of the cyst, existence of complications and engagement of the adjacent structures including the pancreatic and common bile ducts. Furthermore, surgeon’s experience, patient’s age and associated comorbid conditions are also important factors in guiding the surgical approach. The main objectives of the surgical treatment are complete removal of all parasitic elements, avoidance of spillage of the contents of the cyst, and removal of the cyst with maximum tissue conservation of the affected organ. Regarding post-operative follow-up, the patient was monitored by periodic abdominal ultrasound for any possible recurrences, accompanied by routine evaluation of full blood cell count and liver enzymes performed at the start of a 28-day cycle and every 2 weeks during therapy for 4 months to monitor for albendazole toxicity.

## CONCLUSION

PHC is a rare condition, often confused with cystic pancreatic neoplasms. Although PHC presents a unique challenge in pre-operative diagnosis, it should always be considered in the differential diagnosis of cystic pancreatic lesions from endemic areas. Awareness of this phenomenon helps prevent misdiagnosis and inappropriate treatment.
